# Oil Spillage and Heavy Metals Toxicity Risk in the Niger Delta, Nigeria

**DOI:** 10.5696/2156-9614-8.19.180905

**Published:** 2018-08-21

**Authors:** Enegide Chinedu, Chukwuma Kelechukwu Chukwuemeka

**Affiliations:** 1 Department of Pharmacology and Toxicology, Faculty of Pharmaceutical Sciences, University of Nigeria, Nsukka, Nigeria; 2 Department of Plant Science and Biotechnology, Faculty of Biological Sciences, University of Nigeria, Nsukka, Nigeria

**Keywords:** oil spillage, heavy metals, toxicity, Niger Delta

## Abstract

**Background.:**

Heavy metals are known to elicit toxic effects which negatively affect human health. Crude oil is known to contain heavy metals and oil spills contaminate the environment and can result in human exposures to heavy metals. As the seat of crude oil activities, the Niger Delta region experiences the highest rate of oil spills in Nigeria.

**Objective.:**

The purpose of this study was to estimate the quantity of oil spilled into the Niger Delta region from 1976 through 2014 and to assess the resulting heavy metals exposures of the local population.

**Methods.:**

Secondary data from governmental and non-governmental bodies were analyzed and the amount of oil spilled in the Niger Delta region from 1976 through 2014 was determined. The heavy metals present in crude oil from this region and the quantities released into the environment within the study period due to spillage were then extrapolated.

**Results.:**

The Niger Delta region is continuously exposed to a higher rate of oil spills, and about 3.1 million barrels of crude oil enriched in manganese (Mn), iron (Fe), copper (Cu), zinc (Zn), lead (Pb), nickel (Ni), cobalt (Co), cadmium (Cd) and chromium (Cr) were spilled from 1976 to 2014 in this region. Therefore, the occupants of this region may be at risk of heavy metals toxicity.

**Conclusion.:**

To minimize heavy metals exposure and toxicity in the Niger Delta region, effective strategies must be adopted to reduce oil spills. In addition, curtailment and remediation of oil spills should be more rapid.

**Competing Interests.:**

The authors declare no competing financial interests

## Introduction

Heavy metals are metallic elements with a relative density at least five times that of water.^1^ These elements have several toxic effects in humans, and their toxicity is inter-related with their heaviness.^1^ Recently, there has been an increase in ecological and global health concern regarding environmental contamination by heavy metals.^2^ Although these metals occur naturally and are found throughout the earth's crust at low quantities, human exposure generally results from anthropogenic activities such as smelting, mining, and agricultural and industrial activities.^3–5^ Once released into the environment, heavy metals may be taken into the body by inhalation and ingestion. Rapid accumulation in body tissues greater than the detoxification pathways in the body can handle causes a gradual build-up of these metals.^6^,^7^

Oil spills are a source of heavy metal contamination of aquatic and terrestrial environments, especially in oil-producing regions.^8^ Crude oil is a complex mixture of hydrocarbon and non-hydrocarbon compounds (including heavy metals) found in subsurface deposits worldwide. Oil spillage results in release of oil into the natural environment and is associated with activities such as extraction, refining, transportation and storage of crude oil. Spillage also results from accidents, lack of maintenance of engineering equipment and deliberate acts (including oil bunkering and sabotage). In addition, oil spills can also occur as a result of natural disasters such as earthquakes and hurricanes.^8^ Because crude oil contains heavy metals, contamination of the environment with heavy metals is associated with oil spillage. The Niger Delta region experiences a high number of oil spill incidents because it is the seat of crude oil activities in Nigeria.

**Abbreviations**
*WHO*: World Health Organization

The Niger Delta, a fan-shaped area with a landmass of approximately 70,000 km^2^ is located in the southern part of Nigeria and consists of 2 rivers, the Benue River and the Niger River that ultimately drain into the Atlantic Ocean. Its ecology is characterized by a very large floodplain created by the deposition and accumulation of sediments washed down from the Benue and Niger rivers.^9^,^10^ The Niger Delta region forms about 7.5% of the total land mass of Nigeria, and it is inhabited by over 25 million people in 186 Local Government Areas in nine southern states of Nigeria.^9^,^11^ History shows that oil was first discovered in commercial quantities in the Niger Delta in 1956. Since then, the region has continually suffered the negative environmental impacts of oil development.^11^ These negative impacts are due mainly to oil spills in this region. Previous reports indicate that about 50% of oil spills in the Niger Delta result from pipeline corrosion and tanker accidents; other significant causes include operational error, mechanical failure, sabotage and terrorism.^8^,^11^ Nigeria is Africa's main producer of oil, and it has the second largest oil reserve in Africa.^12^ Conservative estimates have shown that Nigeria's oil reserve is about 35 billion barrels; this means that oil activities in the Niger Delta may continue for at least 40 years.^13^

Each year, several post-impact assessment studies are carried out to assess the impact of hazards caused by oil activities and spills on the physical and social environments. Several of these studies have reported the negative socioeconomic impacts of oil spills, such as a decrease in agricultural productivity due to farmland degradation, pollution of traditional fishing grounds and destruction of aquatic life, as well as negative effects on soils, forests and water bodies.^8^,^13^,^14^ However, few of these studies have focused on the health hazards (both immediate and long-term) of oil contamination of the environment and the implications for the inhabitants of the affected communities.^15^ Crude oil contamination has multiple adverse health effects, most of which are due to heavy metal toxicity. The purpose of this study was to evaluate the quantity of oil spilled into the Niger Delta region from 1976 through 2014 and the resulting exposure of occupants to heavy metals.

## Methods

In order to estimate the volume of oil spilled in the Niger Delta region within the study period, data were obtained from reports published by governmental and non-governmental organizations. Secondary data from a United Nations Environment Programme report, an Amnesty International report, a Nigerian Department of Petroleum Resources report, seminar and conference papers, and other published materials were also used.^8^,^16–22^ The quantity of oil spilled in the Niger Delta region from 1976 through 2014, the annual number of oil spill incidents, and the trend of oil spill incidents were recorded. All the obtained data were analyzed descriptively. The quantities of heavy metals released into the Niger Delta through oil spillage were extrapolated based on the individual average concentrations (in crude oil from the region) as reported by Ahmad et al. and the total volume of oil spilled within the study period.^22^

## Results

**Figure 1:**
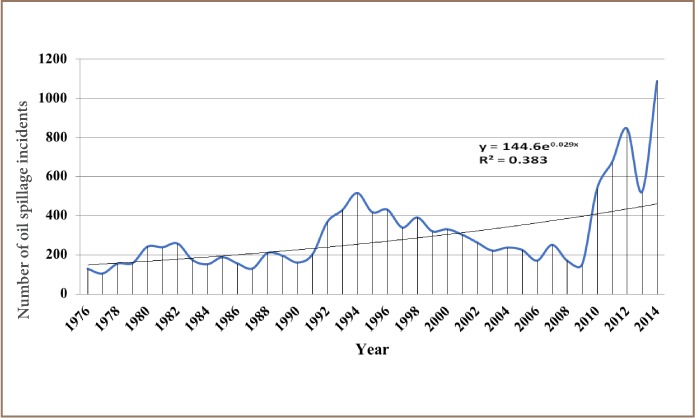
Trend of oil spillage incidents in Nigeria from 1976 – 2014

**Figure 2:**
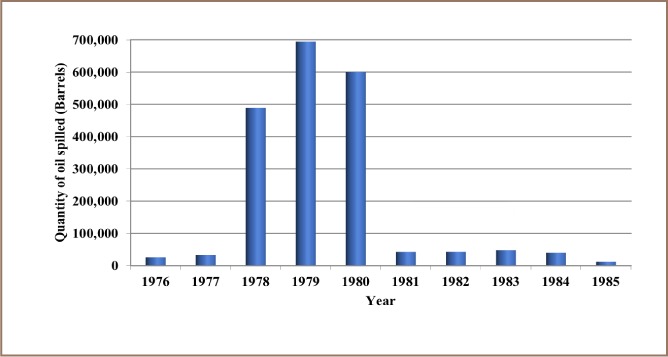
Quantity of oil spilled into the Niger Delta region annually from 1976 – 1985

**Figure 3:**
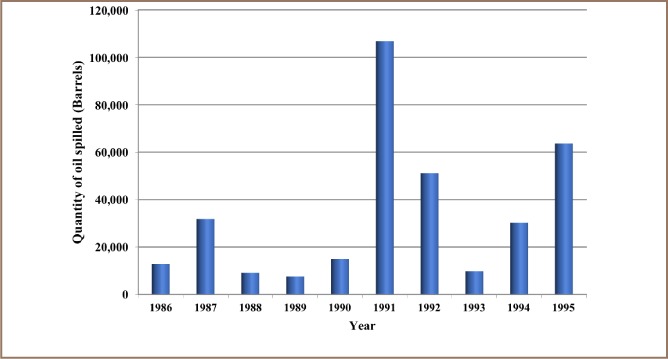
Quantity of oil spilled into the Niger Delta region annually from 1986 – 1995

**Figure 4:**
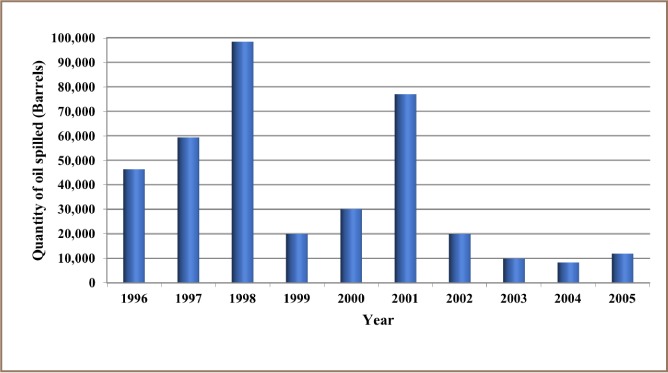
Quantity of oil spilled into the Niger Delta region annually from 1996 – 2005

**Figure 5:**
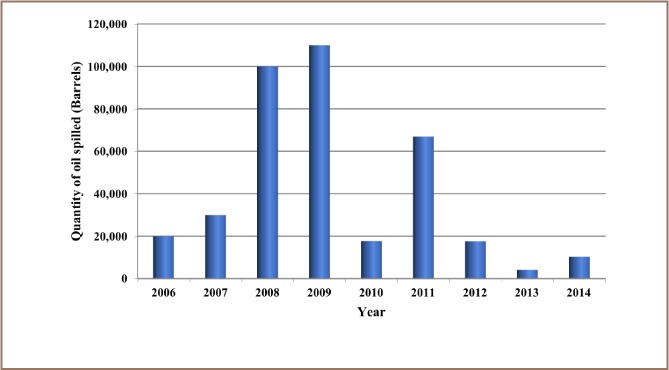
Quantity of oil spilled into the Niger Delta region annually from 2006 – 2014

**Figure 6:**
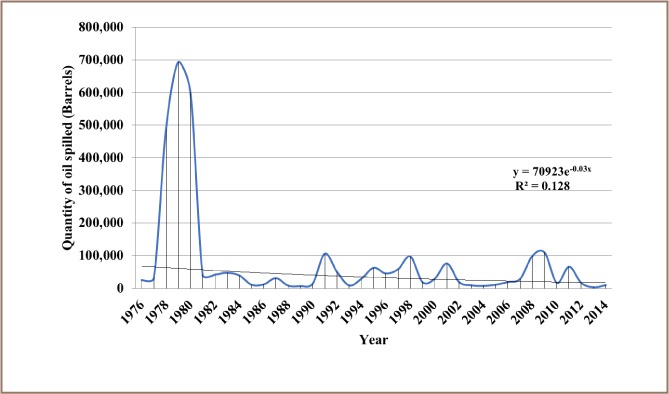
Trend in the annual volume of oil spillage in Nigeria from 1976 – 2014

**Figure 7:**
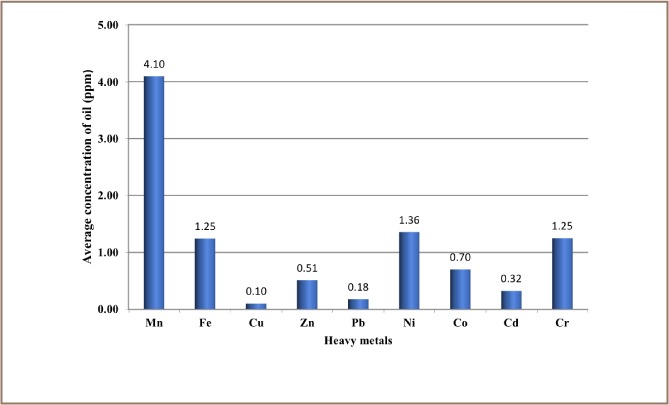
Average concentration of different heavy metals in crude oil from the Niger Delta region

**Figure 8:**
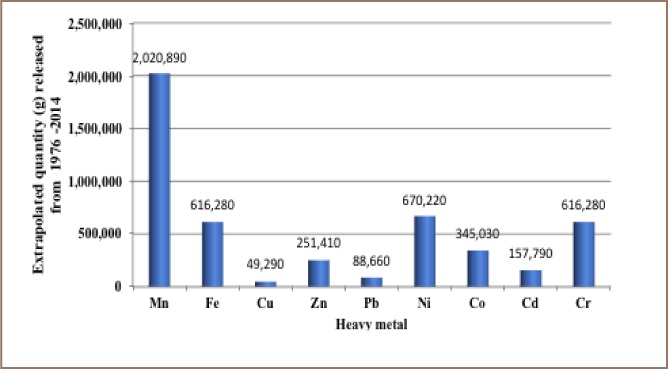
Extrapolated quantity of heavy metals released into the Niger Delta environment from 1976 - 2014 due to oil spillage

The results from the present study show that 12,000 recorded oil spill incidents occurred from 1976 to 2014. The trend in oil spill incidents from 1976 to 2009 did not follow a regular pattern, as there were substantial yearly increases preceded by a decrease within this period *(Figure 1).* However, from 2010 to 2014, there was a substantial increase in oil spill incidents without a corresponding decrease, as 2014 had the highest number of oil spillage incidents within the study period *(Figure 1)*. The data also showed that from 1976 to 2014, about 3.1 million barrels of oil were spilled in the Niger Delta *(Figures 2–5)*. However, the trend for the quantity of oil spilled annually within the study period did not correlate with the trend of annual oil spillage incidents, as there seemed to be a substantial decrease in the volume of oil spilled annually in the Niger Delta *(Figure 6*). From the data obtained, the heavy metals present in oil found in this region include manganese (Mn), iron (Fe), copper (Cu), zinc (Zn), lead (Pb), nickel (Ni), cobalt (Co), cadmium (Cd) and chromium (Cr). The average concentration of Mn was higher compared to the average concentrations of the other heavy metals present in crude oil *(Figure 7)*. The average concentration of Mn in oil from this region was about 4.1 ppm, which is equivalent to 0.6519 g/bbl, hence the extrapolated quantity of Mn released into the Niger Delta environment through oil spillage is about 2,020,890 grams *(Figure 8)*. The average quantity of Fe in oil from this region was 1.25 ppm, equivalent to 0.1988 g/bbl, and the extrapolated quantity released into the Niger Delta environment from 1976 to 2014 was about 616,280 grams *(Figure 8)*. The extrapolated quantity of Cu, Zn, Pb, Ni, Co, Cd and Cr released into the Niger Delta environment due to oil spillage was 49,290, 251,410, 88,660, 670,220, 345,030, 157,790 and 616,280 grams, respectively *(Figure 8)*.

## Discussion

Since the discovery of oil in Nigeria in 1956, the oil sector has grown to be a dominant component of Nigeria's economy.^17^ In 2004, with an average exportation rate of 2.5 million barrels of crude oil per day, the oil and gas sector accounted for about 80% of total government revenue and about 90–95% of export revenues.^23^,^24^ Nigeria is among the world's top ten oil producing nations, and it is the leading oil producing nation in Africa.^25^ This has, however, come at a great price to the Niger Delta region, where virtually all of the oil production in Nigeria takes place, and experiences a higher rate of oil spills. Oil contains several types of heavy metals with dangerous health effects; hence oil spills result in environmental contamination with heavy metals and heavy metal toxicity, posing a health risk to residents of this region.

A system for the accurate reporting of oil spills in the Niger Delta remains elusive. Thus, information is incomplete on the number of oil spillage incidents and the quantity of crude oil spilled in this region.^11^,^26^ This deficiency may be ascribed to several factors, including impassable roads, reporting delays, security problems/violence, and governmental negligence/oversight. Hence, the available data may not fully reflect the actual incidence rate and volume of oil spilled in the Niger Delta region.^27^ Despite this, the available data is valuable for estimating the exposure of the occupants of this region to the detrimental health effects of oil spillage.

The data obtained in the present study indicate that this region is constantly exposed to oil spills and that there has been a substantial increase in the annual occurrence of oil spill incidents in the Niger Delta region *(Figure 1)*.

This indicates that the measures or strategies adopted to reduce oil spillage incidents have not been effective. The reasons for this may include non-implementation of incident prevention strategies or the adoption of the wrong strategy for this purpose. In any case, an alarming number of oil spill incidents (12,000) were recorded from 1976 to 2014 *(Figure 1)*. These spills occurred through diverse mechanisms such as tanker accidents, pipeline corrosion, sabotage, machine failure, inability to effectively control wells, and oil bunkering.^8^,^11^,^28^ However, the annual volume of oil spilled followed a decreasing trend *(Figure 6)*. Although the annual incidence increased, the annual volume of spillage decreased considerably, in contrast with the annual oil spillage incidence trend, which also showed an increase *(Figures 1–6)*. This may be because an effective mechanism was put in place for a quick response to reported incidents, thereby preventing the loss of larger quantities of oil. In addition, a deficiency in the accurate reporting of oil spill incidence and volume of oil spilled may also be a factor contributing to this phenomenon.

Although the quantities of these heavy metals differ slightly across different oil wells (onshore and offshore), the average quantity of Mn exceeded that of other heavy metals in crude oil from this region *(Figure 7)*; hence the extrapolated quantity of Mn released within the study period also tended to be higher *(Figure 8)*.^22^ Generally, at toxic concentrations these metals can elicit detrimental effects such as malaise, nausea, cough, headache, diarrhea, sore throat, sore eyes, itchy skin and skin rash.^29^ However, each heavy metal causes specific harmful effects in humans at toxic levels. This ranges from mild effects (e.g. upset stomach) to potentially fatal toxic effects that may lead to organ impairment/damage and mortality.^30–36^

Our findings show that the Niger Delta region may be contaminated with substantial amounts of these heavy metals due to oil spills. Hence the inhabitants of this region, in addition to other socioeconomic challenges, may also be at risk of heavy metals toxicity. Tchounwou et al. and Wang et al. found that co-exposure to more than one heavy metal can produce effects that are more dangerous to human health than those caused by exposure to individual heavy metals.^2^,^37^ Thus, residents of the Niger Delta region may be at risk of co-exposures produced by oil spills. There are several mechanisms by which metals from contaminated aquatic and terrestrial environments can enter the human body, including eating contaminated farm products, fish from contaminated ponds/rivers, and untreated cooking and drinking water from contaminated sources. Most of the residents of this region are unaware of the potential health threats posed by heavy metals due to oil spills. In addition, due to poor access to portable water by the majority of the occupants in this region, it is almost impossible for them to avoid cooking with or drinking water from contaminated sources even when they understand the risks.

Comprehensive data on the concentration of heavy metals in both aquatic and terrestrial environments of the Niger Delta are currently unavailable; however, studies have been carried out to evaluate the concentration of different heavy metals in a few locations/communities in this area. Studies by Ogamba et al. evaluating the level of heavy metals in water and sediments from Kolo Creek and Oribhabor et al. which evaluated the concentration of heavy metals in surface water from Buguma Creek reported that the concentrations of heavy metals were within permissible limits specified by the World Health Organization (WHO).^38^,^39^,^40^ Ubiogoro and Adeyemo evaluated water and fish samples from the Gbokodo river in Warri, the River Ethiope in Sapele, the Urie River in Igbide Isoko, Asaba-Ase creek, the Aragba River in Abraka, and Uzere creek, and found that most heavy metals were within the recommended limits set by the WHO, except for Fe and Ni, which were higher than the recommended level in fish.^41^

However, a study by Owamah which evaluated the concentration of heavy metals (Pb, Ni, Cu, Cr, Fe, Co, Cd, and mercury (Hg)) in surface water from the River Ijana, Warri showed that the concentrations of heavy metals were higher than the WHO recommended limits, with Pb having the highest concentration.^42^ In addition, studies by Emuedo et al. which assessed the concentration of heavy metals (Cr, Zn, Cu, Cd and Pb) in water samples from Nembe, Okirika and Okpare communities; and Ejike et al. which evaluated concentrations of heavy metals (Pb, As and Cd) in underground water samples from the Umuebulu community revealed that they were present in concentrations above the WHO recommended limits for drinking water.^43^,^44^ A study by Gideon-Ogero on cassava harvested from Afiesere, Ekiugbo, Orogun and Ofoma communities revealed that Pb was present above the WHO recommended limit.^45^,^46^ Muawiya et al. reported that Mn, Ni, Pb and Cd were present in croaker fish from Bonny and Finima communities in concentrations which exceeded the maximum permissible limits recommended by the WHO for fish; while Okoye and Okwute also reported high concentrations of Pb in cassava tubers and plantain fruits from the Olomo community above the WHO acceptable limits for food.^46–48^ Symptoms including abdominal pain, nausea, vomiting, body pain, headache, dizziness and depression were also reportedly prevalent among the local population.^48^

The findings from our study and the other studies we have highlighted suggests that although a large quantity of crude oil has been released into the Niger Delta region, the concentration of heavy metals in the terrestrial and aquatic environments may vary across the different communities in this region. The concentration of heavy metals in the aquatic and terrestrial environments in some communities may be within allowable limits, but may be above allowable limits in others, elevating the risk of toxicity. However, a holistic assessment of the concentration of heavy metals is needed in this region to identify communities at risk of heavy metals toxicity and corresponding risk levels.

## Conclusions

The present study shows that the occupants of the Niger Delta region may be at risk of heavy metal toxicity because of the large amount of oil being spilled in the region. To minimize the level of exposure to heavy metals and the risk of toxicity in the Niger Delta region, effective strategies should be adopted to reduce the occurrence of oil spills. Curtailment and remediation of oil spills should be more rapid. In addition, due to the wide range of detrimental health effects that heavy metals can elicit in humans, the government should establish a center where the local population can be assessed and de-toxified of heavy metals as necessary. A holistic assessment of the risk level of heavy metal toxicity in the Niger Delta is also recommended.
